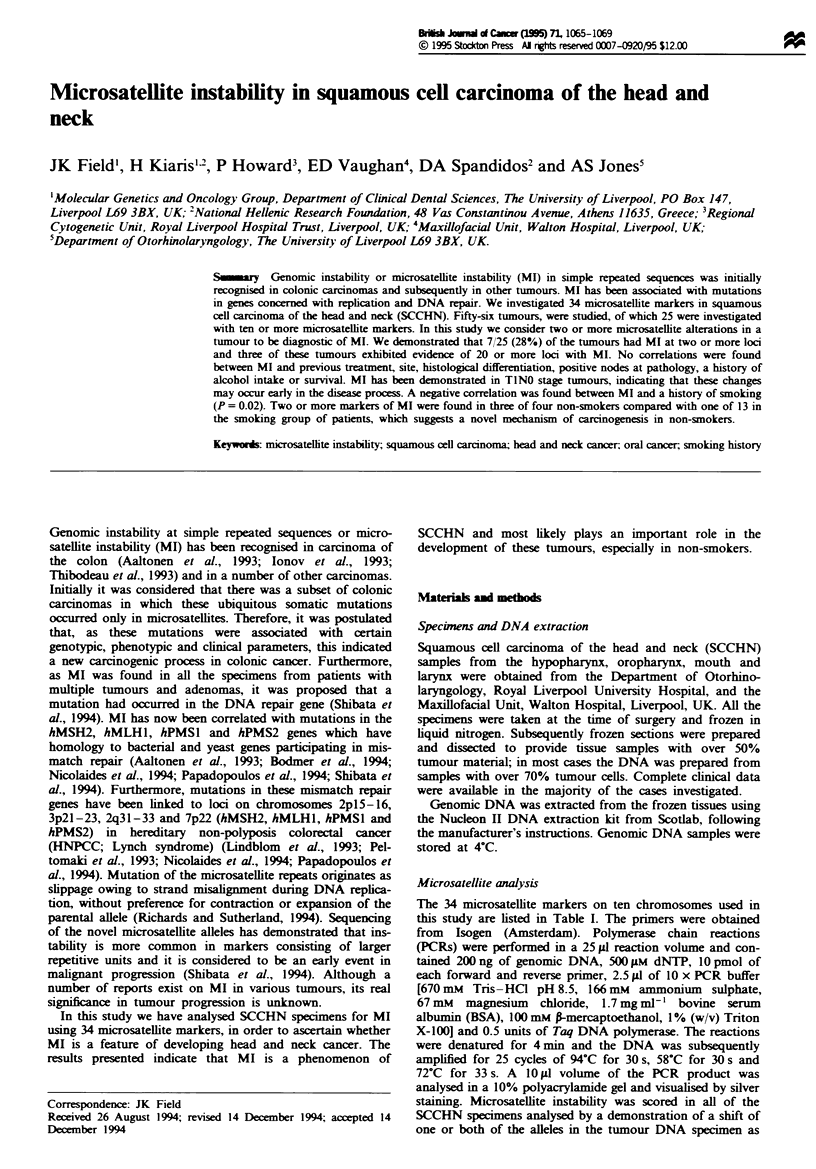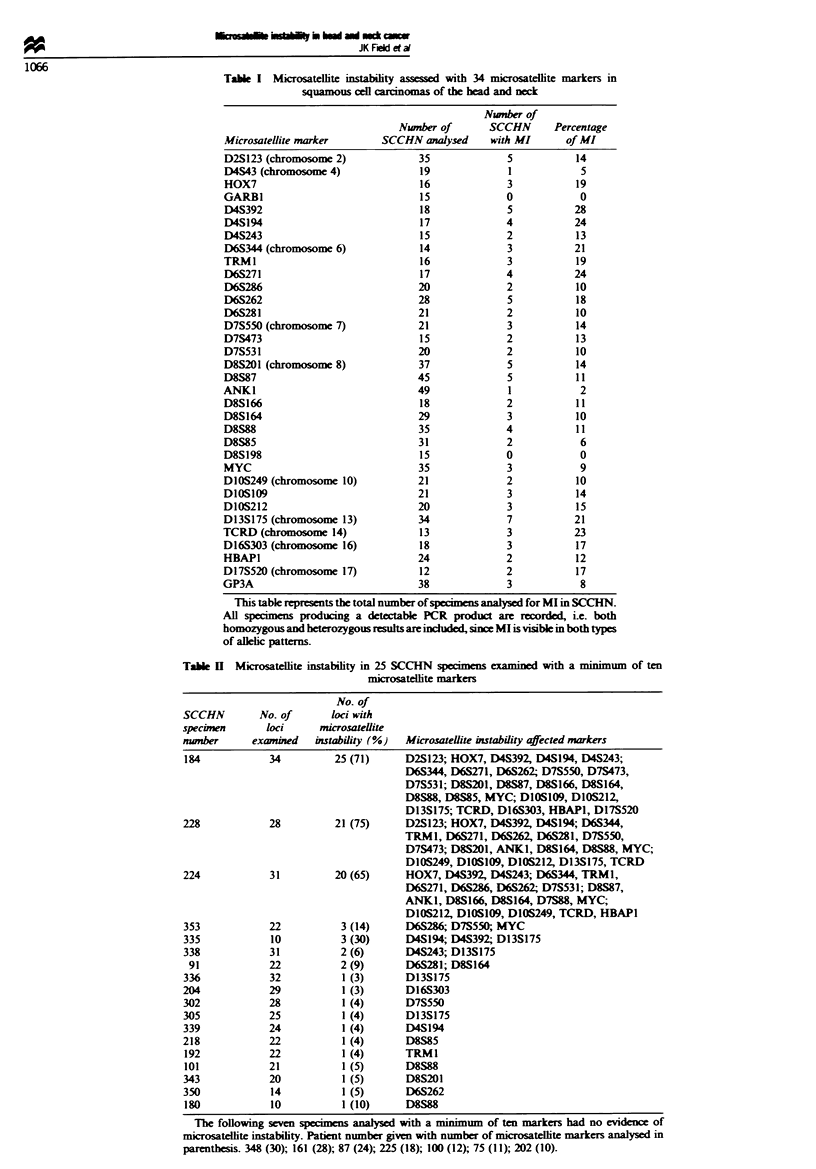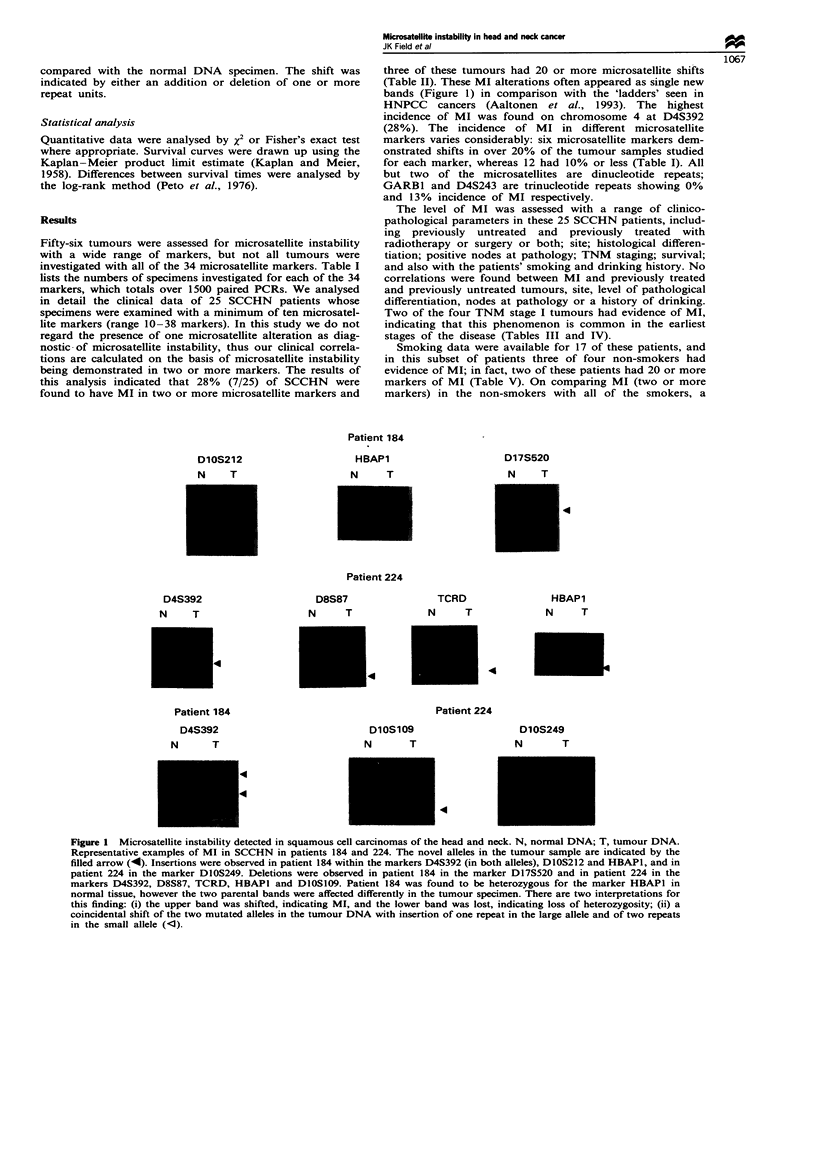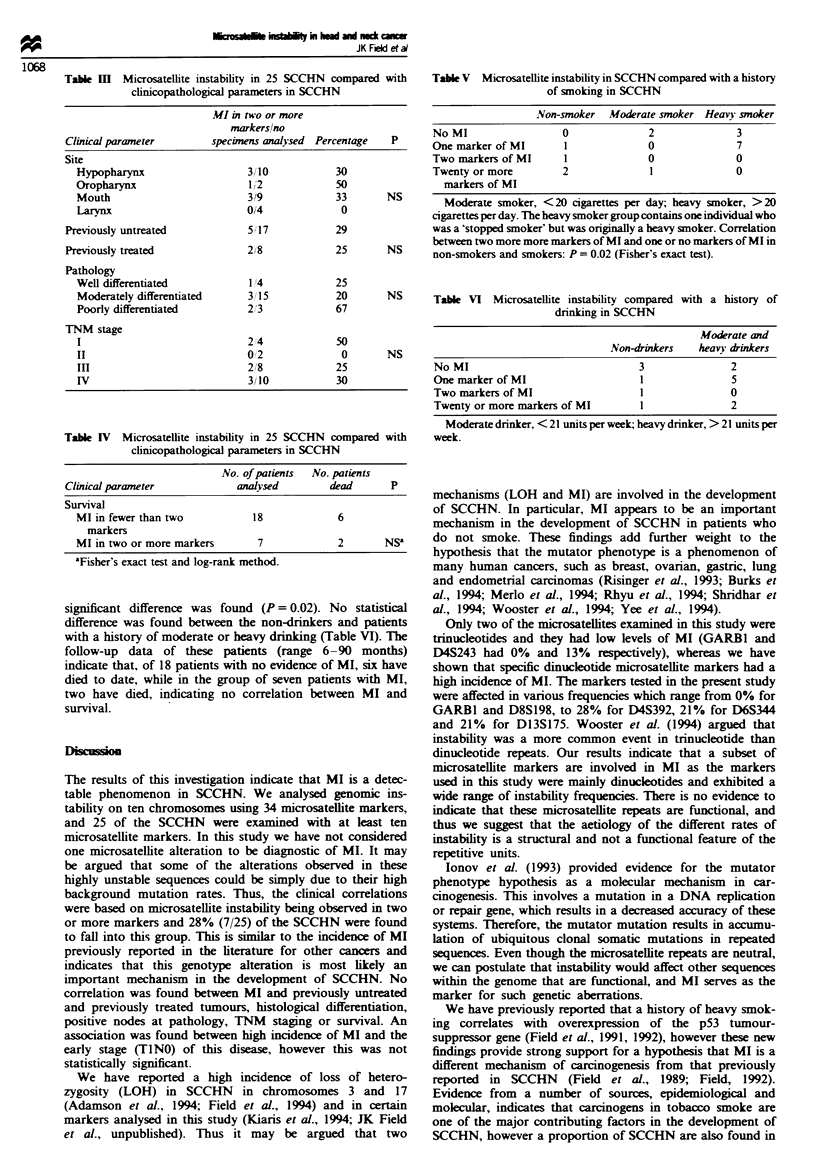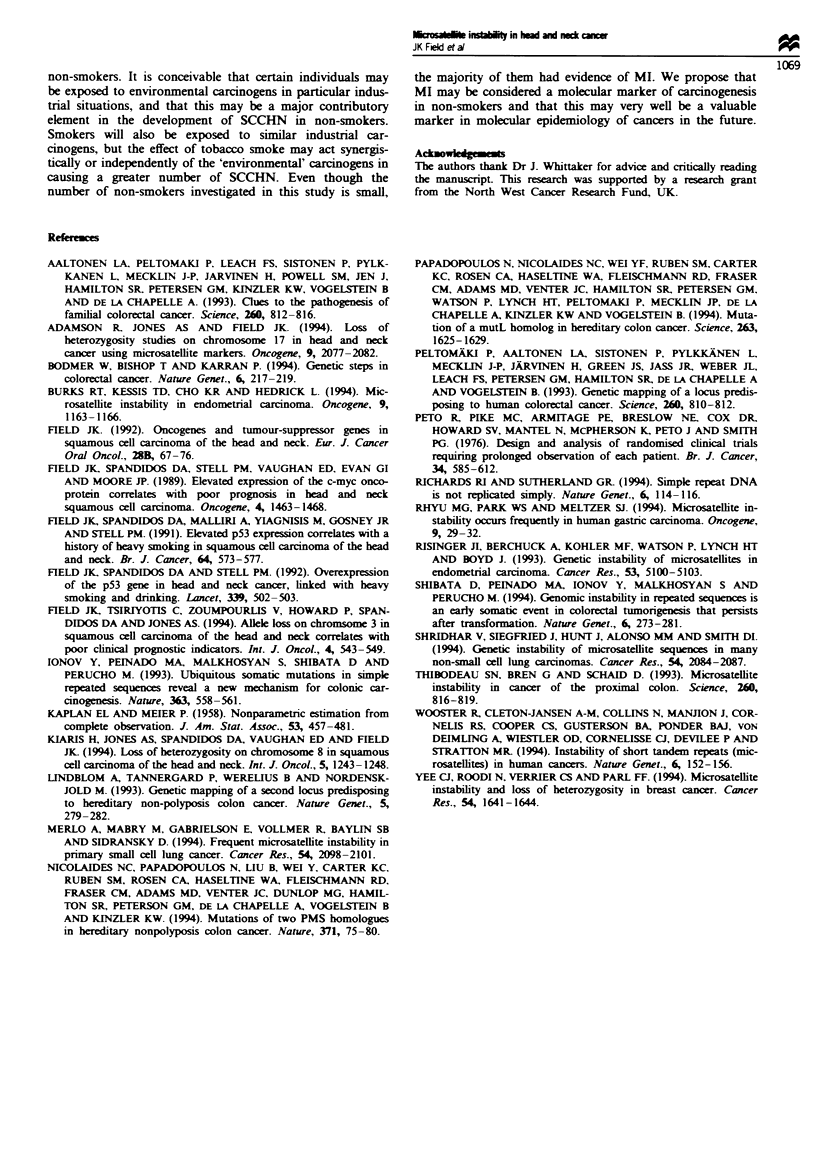# Microsatellite instability in squamous cell carcinoma of the head and neck.

**DOI:** 10.1038/bjc.1995.205

**Published:** 1995-05

**Authors:** J. K. Field, H. Kiaris, P. Howard, E. D. Vaughan, D. A. Spandidos, A. S. Jones

**Affiliations:** Department of Clinical Dental Sciences, University of Liverpool, UK.

## Abstract

**Images:**


© 1995 Stockton Press All rights reserved 0007-0920/95 $12.00

### Microsatellite instability in squamous cell carcinoma of the head and neck

JK Field[1], H Kiaris[1,2], P Howard[3], ED Vaughan[4], DA Spandidos[2] and AS Jones[5]

[1]*Molecular Genetics and Oncology Group, Department of Clinical Dental Sciences, The University of Liverpool, PO Box 147, Liverpool L69 3BX, UK;* [2]*National Hellenic Research Foundation, 48 Vas Constantinou Avenue, Athens 11635, Greece;* [3]*Regional Cytogenetic Unit, Royal Liverpool Hospital Trust, Liverpool, UK;* [4]*Maxillofacial Unit, Walton Hospital, Liverpool, UK;* [5]*Department of Otorhinolaryngology, The University of Liverpool L69 3BX, UK.*

**Summary** Genomic instability or microsatellite instability (MI) in simple repeated sequences was initially recognised in colonic carcinomas and subsequently in other tumours. MI has been associated with mutations in genes concerned with replication and DNA repair. We investigated 34 microsatellite markers in squamous cell carcinoma of the head and neck (SCCHN). Fifty-six tumours, were studied, of which 25 were investigated with ten or more microsatellite markers. In this study we consider two or more microsatellite alterations in a tumour to be diagnostic of MI. We demonstrated that 7/25 (28%) of the tumours had MI at two or more loci and three of these tumours exhibited evidence of 20 or more loci with MI. No correlations were found between MI and previous treatment, site, histological differentiation, positive nodes at pathology, a history of alcohol intake or survival. MI has been demonstrated in T1N0 stage tumours, indicating that these changes may occur early in the disease process. A negative correlation was found between MI and a history of smoking ($P = 0.02$). Two or more markers of MI were found in three of four non-smokers compared with one of 13 in the smoking group of patients, which suggests a novel mechanism of carcinogenesis in non-smokers.

**Keywords**: microsatellite instability; squamous cell carcinoma; head and neck cancer; oral cancer; smoking history

Genomic instability at simple repeated sequences or microsatellite instability (MI) has been recognised in carcinoma of the colon (Aaltonen *et al.*, 1993; Ionov *et al.*, 1993; Thibodeau *et al.*, 1993) and in a number of other carcinomas. Initially it was considered that there was a subset of colonic carcinomas in which these ubiquitous somatic mutations occurred only in microsatellites. Therefore, it was postulated that, as these mutations were associated with certain genotypic, phenotypic and clinical parameters, this indicated a new carcinogenic process in colonic cancer. Furthermore, as MI was found in all the specimens from patients with multiple tumours and adenomas, it was proposed that a mutation had occurred in the DNA repair gene (Shibata *et al.*, 1994). MI has now been correlated with mutations in the *h*MSH2, *h*MLH1, *h*PMS1 and *h*PMS2 genes which have homology to bacterial and yeast genes participating in mismatch repair (Aaltonen *et al.*, 1993; Bodmer *et al.*, 1994; Nicolaides *et al.*, 1994; Papadopoulos *et al.*, 1994; Shibata *et al.*, 1994). Furthermore, mutations in these mismatch repair genes have been linked to loci on chromosomes 2p15-16, 3p21-23, 2q31-33 and 7p22 (*h*MSH2, *h*MLH1, *h*PMS1 and *h*PMS2) in hereditary non-polyposis colorectal cancer (HNPCC; Lynch syndrome) (Lindblom *et al.*, 1993; Peltomaki *et al.*, 1993; Nicolaides *et al.*, 1994; Papadopoulos *et al.*, 1994). Mutation of the microsatellite repeats originates as slippage owing to strand misalignment during DNA replication, without preference for contraction or expansion of the parental allele (Richards and Sutherland, 1994). Sequencing of the novel microsatellite alleles has demonstrated that instability is more common in markers consisting of larger repetitive units and it is considered to be an early event in malignant progression (Shibata *et al.*, 1994). Although a number of reports exist on MI in various tumours, its real significance in tumour progression is unknown.

In this study we have analysed SCCHN specimens for MI using 34 microsatellite markers, in order to ascertain whether MI is a feature of developing head and neck cancer. The results presented indicate that MI is a phenomenon of SCCHN and most likely plays an important role in the development of these tumours, especially in non-smokers.

Correspondence: JK Field
Received 26 August 1994; revised 14 December 1994; accepted 14 December 1994

### Materials and methods

### *Specimens and DNA extraction*

Squamous cell carcinoma of the head and neck (SCCHN) samples from the hypopharynx, oropharynx, mouth and larynx were obtained from the Department of Otorhinolaryngology, Royal Liverpool University Hospital, and the Maxillofacial Unit, Walton Hospital, Liverpool, UK. All the specimens were taken at the time of surgery and frozen in liquid nitrogen. Subsequently frozen sections were prepared and dissected to provide tissue samples with over 50% tumour material; in most cases the DNA was prepared from samples with over 70% tumour cells. Complete clinical data were available in the majority of the cases investigated.

Genomic DNA was extracted from the frozen tissues using the Nucleon II DNA extraction kit from Scotlab, following the manufacturer's instructions. Genomic DNA samples were stored at 4°C.

### *Microsatellite analysis*

The 34 microsatellite markers on ten chromosomes used in this study are listed in Table I. The primers were obtained from Isogen (Amsterdam). Polymerase chain reactions (PCRs) were performed in a 25 µl reaction volume and contained 200 ng of genomic DNA, 500 µM dNTP, 10 pmol of each forward and reverse primer, 2.5 µl of 10 × PCR buffer [670 mM Tris-HCl pH 8.5, 166 mM ammonium sulphate, 67 mM magnesium chloride, 1.7 mg ml$^{-1}$ bovine serum albumin (BSA), 100 mM β-mercaptoethanol, 1% (w/v) Triton X-100] and 0.5 units of *Taq* DNA polymerase. The reactions were denatured for 4 min and the DNA was subsequently amplified for 25 cycles of 94°C for 30 s, 58°C for 30 s and 72°C for 33 s. A 10 µl volume of the PCR product was analysed in a 10% polyacrylamide gel and visualised by silver staining. Microsatellite instability was scored in all of the SCCHN specimens analysed by a demonstration of a shift of one or both of the alleles in the tumour DNA specimen as

**Table I** Microsatellite instability assessed with 34 microsatellite markers in squamous cell carcinomas of the head and neck

| *Microsatellite marker* | *Number of SCCHN analysed* | *Number of SCCHN with MI* | *Percentage of MI* |
|---|---|---|---|
| D2S123 (chromosome 2) | 35 | 5 | 14 |
| D4S43 (chromosome 4) | 19 | 1 | 5 |
| HOX7 | 16 | 3 | 19 |
| GARB1 | 15 | 0 | 0 |
| D4S392 | 18 | 5 | 28 |
| D4S194 | 17 | 4 | 24 |
| D4S243 | 15 | 2 | 13 |
| D6S344 (chromosome 6) | 14 | 3 | 21 |
| TRM1 | 16 | 3 | 19 |
| D6S271 | 17 | 4 | 24 |
| D6S286 | 20 | 2 | 10 |
| D6S262 | 28 | 5 | 18 |
| D6S281 | 21 | 2 | 10 |
| D7S550 (chromosome 7) | 21 | 3 | 14 |
| D7S473 | 15 | 2 | 13 |
| D7S531 | 20 | 2 | 10 |
| D8S201 (chromosome 8) | 37 | 5 | 14 |
| D8S87 | 45 | 5 | 11 |
| ANK1 | 49 | 1 | 2 |
| D8S166 | 18 | 2 | 11 |
| D8S164 | 29 | 3 | 10 |
| D8S88 | 35 | 4 | 11 |
| D8S85 | 31 | 2 | 6 |
| D8S198 | 15 | 0 | 0 |
| MYC | 35 | 3 | 9 |
| D10S249 (chromosome 10) | 21 | 2 | 10 |
| D10S109 | 21 | 3 | 14 |
| D10S212 | 20 | 3 | 15 |
| D13S175 (chromosome 13) | 34 | 7 | 21 |
| TCRD (chromosome 14) | 13 | 3 | 23 |
| D16S303 (chromosome 16) | 18 | 3 | 17 |
| HBAP1 | 24 | 2 | 12 |
| D17S520 (chromosome 17) | 12 | 2 | 17 |
| GP3A | 38 | 3 | 8 |

This table represents the total number of specimens analysed for MI in SCCHN. All specimens producing a detectable PCR product are recorded, i.e. both homozygous and heterozygous results are included, since MI is visible in both types of allelic patterns.

**Table II** Microsatellite instability in 25 SCCHN specimens examined with a minimum of ten microsatellite markers

| *SCCHN specimen number* | *No. of loci examined* | *No. of loci with microsatellite instability (%)* | *Microsatellite instability affected markers* |
|---|---|---|---|
| 184 | 34 | 25 (71) | D2S123; HOX7, D4S392, D4S194, D4S243; D6S344, D6S271, D6S262; D7S550, D7S473, D7S531; D8S201, D8S87, D8S166, D8S164, D8S88, D8S85, MYC; D10S109, D10S212, D13S175; TCRD, D16S303, HBAP1, D17S520 |
| 228 | 28 | 21 (75) | D2S123; HOX7, D4S392, D4S194; D6S344, TRM1, D6S271, D6S262, D6S281, D7S550, D7S473; D8S201, ANK1, D8S164, D8S88, MYC; D10S249, D10S109, D10S212, D13S175, TCRD |
| 224 | 31 | 20 (65) | HOX7, D4S392, D4S243; D6S344, TRM1, D6S271, D6S286, D6S262; D7S531; D8S87, ANK1, D8S166, D8S164, D7S88, MYC; D10S212, D10S109, D10S249, TCRD, HBAP1 |
| 353 | 22 | 3 (14) | D6S286; D7S550; MYC |
| 335 | 10 | 3 (30) | D4S194; D4S392; D13S175 |
| 338 | 31 | 2 (6) | D4S243; D13S175 |
| 91 | 22 | 2 (9) | D6S281; D8S164 |
| 336 | 32 | 1 (3) | D13S175 |
| 204 | 29 | 1 (3) | D16S303 |
| 302 | 28 | 1 (4) | D7S550 |
| 305 | 25 | 1 (4) | D13S175 |
| 339 | 24 | 1 (4) | D4S194 |
| 218 | 22 | 1 (4) | D8S85 |
| 192 | 22 | 1 (4) | TRM1 |
| 101 | 21 | 1 (5) | D8S88 |
| 343 | 20 | 1 (5) | D8S201 |
| 350 | 14 | 1 (5) | D6S262 |
| 180 | 10 | 1 (10) | D8S88 |

The following seven specimens analysed with a minimum of ten markers had no evidence of microsatellite instability. Patient number given with number of microsatellite markers analysed in parenthesis. 348 (30); 161 (28); 87 (24); 225 (18); 100 (12); 75 (11); 202 (10).

compared with the normal DNA specimen. The shift was indicated by either an addition or deletion of one or more repeat units.

*Statistical analysis*

Quantitative data were analysed by $\chi^2$ or Fisher's exact test where appropriate. Survival curves were drawn up using the Kaplan–Meier product limit estimate (Kaplan and Meier, 1958). Differences between survival times were analysed by the log-rank method (Peto *et al.*, 1976).

### Results

Fifty-six tumours were assessed for microsatellite instability with a wide range of markers, but not all tumours were investigated with all of the 34 microsatellite markers. Table I lists the numbers of specimens investigated for each of the 34 markers, which totals over 1500 paired PCRs. We analysed in detail the clinical data of 25 SCCHN patients whose specimens were examined with a minimum of ten microsatellite markers (range 10–38 markers). In this study we do not regard the presence of one microsatellite alteration as diagnostic of microsatellite instability, thus our clinical correlations are calculated on the basis of microsatellite instability being demonstrated in two or more markers. The results of this analysis indicated that 28% (7/25) of SCCHN were found to have MI in two or more microsatellite markers and three of these tumours had 20 or more microsatellite shifts (Table II). These MI alterations often appeared as single new bands (Figure 1) in comparison with the 'ladders' seen in HNPCC cancers (Aaltonen *et al.*, 1993). The highest incidence of MI was found on chromosome 4 at D4S392 (28%). The incidence of MI in different microsatellite markers varies considerably: six microsatellite markers demonstrated shifts in over 20% of the tumour samples studied for each marker, whereas 12 had 10% or less (Table I). All but two of the microsatellites are dinucleotide repeats; GARB1 and D4S243 are trinucleotide repeats showing 0% and 13% incidence of MI respectively.

The level of MI was assessed with a range of clinicopathological parameters in these 25 SCCHN patients, including previously untreated and previously treated with radiotherapy or surgery or both; site; histological differentiation; positive nodes at pathology; TNM staging; survival; and also with the patients' smoking and drinking history. No correlations were found between MI and previously treated and previously untreated tumours, site, level of pathological differentiation, nodes at pathology or a history of drinking. Two of the four TNM stage I tumours had evidence of MI, indicating that this phenomenon is common in the earliest stages of the disease (Tables III and IV).

Smoking data were available for 17 of these patients, and in this subset of patients three of four non-smokers had evidence of MI; in fact, two of these patients had 20 or more markers of MI (Table V). On comparing MI (two or more markers) in the non-smokers with all of the smokers, a

**Figure 1** Microsatellite instability detected in squamous cell carcinomas of the head and neck. N, normal DNA; T, tumour DNA. Representative examples of MI in SCCHN in patients 184 and 224. The novel alleles in the tumour sample are indicated by the filled arrow (◀). Insertions were observed in patient 184 within the markers D4S392 (in both alleles), D10S212 and HBAP1, and in patient 224 in the marker D10S249. Deletions were observed in patient 184 in the marker D17S520 and in patient 224 in the markers D4S392, D8S87, TCRD, HBAP1 and D10S109. Patient 184 was found to be heterozygous for the marker HBAP1 in normal tissue, however the two parental bands were affected differently in the tumour specimen. There are two interpretations for this finding: (i) the upper band was shifted, indicating MI, and the lower band was lost, indicating loss of heterozygosity; (ii) a coincidental shift of the two mutated alleles in the tumour DNA with insertion of one repeat in the large allele and of two repeats in the small allele (◁).

**Table III** Microsatellite instability in 25 SCCHN compared with clinicopathological parameters in SCCHN

| *Clinical parameter* | *MI in two or more markers/no specimens analysed* | *Percentage* | P |
|---|---|---|---|
| Site | | | |
| Hypopharynx | 3/10 | 30 | |
| Oropharynx | 1/2 | 50 | |
| Mouth | 3/9 | 33 | NS |
| Larynx | 0/4 | 0 | |
| Previously untreated | 5/17 | 29 | |
| Previously treated | 2/8 | 25 | NS |
| Pathology | | | |
| Well differentiated | 1/4 | 25 | |
| Moderately differentiated | 3/15 | 20 | NS |
| Poorly differentiated | 2/3 | 67 | |
| TNM stage | | | |
| I | 2/4 | 50 | |
| II | 0/2 | 0 | NS |
| III | 2/8 | 25 | |
| IV | 3/10 | 30 | |

**Table IV** Microsatellite instability in 25 SCCHN compared with clinicopathological parameters in SCCHN

| *Clinical parameter* | *No. of patients analysed* | *No. patients dead* | P |
|---|---|---|---|
| Survival | | | |
| MI in fewer than two markers | 18 | 6 | |
| MI in two or more markers | 7 | 2 | NS[a] |

[a]Fisher's exact test and log-rank method.

**Table V** Microsatellite instability in SCCHN compared with a history of smoking in SCCHN

| | *Non-smoker* | *Moderate smoker* | *Heavy smoker* |
|---|---|---|---|
| No MI | 0 | 2 | 3 |
| One marker of MI | 1 | 0 | 7 |
| Two markers of MI | 1 | 0 | 0 |
| Twenty or more markers of MI | 2 | 1 | 0 |

Moderate smoker, <20 cigarettes per day; heavy smoker, >20 cigarettes per day. The heavy smoker group contains one individual who was a 'stopped smoker' but was originally a heavy smoker. Correlation between two more more markers of MI and one or no markers of MI in non-smokers and smokers: $P = 0.02$ (Fisher's exact test).

**Table VI** Microsatellite instability compared with a history of drinking in SCCHN

| | *Non-drinkers* | *Moderate and heavy drinkers* |
|---|---|---|
| No MI | 3 | 2 |
| One marker of MI | 1 | 5 |
| Two markers of MI | 1 | 0 |
| Twenty or more markers of MI | 1 | 2 |

Moderate drinker, <21 units per week; heavy drinker, >21 units per week.

significant difference was found ($P = 0.02$). No statistical difference was found between the non-drinkers and patients with a history of moderate or heavy drinking (Table VI). The follow-up data of these patients (range 6–90 months) indicate that, of 18 patients with no evidence of MI, six have died to date, while in the group of seven patients with MI, two have died, indicating no correlation between MI and survival.

### Discussion

The results of this investigation indicate that MI is a detectable phenomenon in SCCHN. We analysed genomic instability on ten chromosomes using 34 microsatellite markers, and 25 of the SCCHN were examined with at least ten microsatellite markers. In this study we have not considered one microsatellite alteration to be diagnostic of MI. It may be argued that some of the alterations observed in these highly unstable sequences could be simply due to their high background mutation rates. Thus, the clinical correlations were based on microsatellite instability being observed in two or more markers and 28% (7/25) of the SCCHN were found to fall into this group. This is similar to the incidence of MI previously reported in the literature for other cancers and indicates that this genotype alteration is most likely an important mechanism in the development of SCCHN. No correlation was found between MI and previously untreated and previously treated tumours, histological differentiation, positive nodes at pathology, TNM staging or survival. An association was found between high incidence of MI and the early stage (T1N0) of this disease, however this was not statistically significant.

We have reported a high incidence of loss of heterozygosity (LOH) in SCCHN in chromosomes 3 and 17 (Adamson *et al.*, 1994; Field *et al.*, 1994) and in certain markers analysed in this study (Kiaris *et al.*, 1994; JK Field *et al.*, unpublished). Thus it may be argued that two mechanisms (LOH and MI) are involved in the development of SCCHN. In particular, MI appears to be an important mechanism in the development of SCCHN in patients who do not smoke. These findings add further weight to the hypothesis that the mutator phenotype is a phenomenon of many human cancers, such as breast, ovarian, gastric, lung and endometrial carcinomas (Risinger *et al.*, 1993; Burks *et al.*, 1994; Merlo *et al.*, 1994; Rhyu *et al.*, 1994; Shridhar *et al.*, 1994; Wooster *et al.*, 1994; Yee *et al.*, 1994).

Only two of the microsatellites examined in this study were trinucleotides and they had low levels of MI (GARB1 and D4S243 had 0% and 13% respectively), whereas we have shown that specific dinucleotide microsatellite markers had a high incidence of MI. The markers tested in the present study were affected in various frequencies which range from 0% for GARB1 and D8S198, to 28% for D4S392, 21% for D6S344 and 21% for D13S175. Wooster *et al.* (1994) argued that instability was a more common event in trinucleotide than dinucleotide repeats. Our results indicate that a subset of microsatellite markers are involved in MI as the markers used in this study were mainly dinucleotides and exhibited a wide range of instability frequencies. There is no evidence to indicate that these microsatellite repeats are functional, and thus we suggest that the aetiology of the different rates of instability is a structural and not a functional feature of the repetitive units.

Ionov *et al.* (1993) provided evidence for the mutator phenotype hypothesis as a molecular mechanism in carcinogenesis. This involves a mutation in a DNA replication or repair gene, which results in a decreased accuracy of these systems. Therefore, the mutator mutation results in accumulation of ubiquitous clonal somatic mutations in repeated sequences. Even though the microsatellite repeats are neutral, we can postulate that instability would affect other sequences within the genome that are functional, and MI serves as the marker for such genetic aberrations.

We have previously reported that a history of heavy smoking correlates with overexpression of the p53 tumour-suppressor gene (Field *et al.*, 1991, 1992), however these new findings provide strong support for a hypothesis that MI is a different mechanism of carcinogenesis from that previously reported in SCCHN (Field *et al.*, 1989; Field, 1992). Evidence from a number of sources, epidemiological and molecular, indicates that carcinogens in tobacco smoke are one of the major contributing factors in the development of SCCHN, however a proportion of SCCHN are also found in non-smokers. It is conceivable that certain individuals may be exposed to environmental carcinogens in particular industrial situations, and that this may be a major contributory element in the development of SCCHN in non-smokers. Smokers will also be exposed to similar industrial carcinogens, but the effect of tobacco smoke may act synergistically or independently of the 'environmental' carcinogens in causing a greater number of SCCHN. Even though the number of non-smokers investigated in this study is small, the majority of them had evidence of MI. We propose that MI may be considered a molecular marker of carcinogenesis in non-smokers and that this may very well be a valuable marker in molecular epidemiology of cancers in the future.

**Acknowledgements**
The authors thank Dr J. Whittaker for advice and critically reading the manuscript. This research was supported by a research grant from the North West Cancer Research Fund, UK.

**References**

AALTONEN LA, PELTOMAKI P, LEACH FS, SISTONEN P, PYLKKANEN L, MECKLIN J-P, JARVINEN H, POWELL SM, JEN J, HAMILTON SR, PETERSEN GM, KINZLER KW, VOGELSTEIN B AND DE LA CHAPELLE A. (1993). Clues to the pathogenesis of familial colorectal cancer. *Science*, **260**, 812–816.
ADAMSON R, JONES AS AND FIELD JK. (1994). Loss of heterozygosity studies on chromosome 17 in head and neck cancer using microsatellite markers. *Oncogene*, **9**, 2077–2082.
BODMER W, BISHOP T AND KARRAN P. (1994). Genetic steps in colorectal cancer. *Nature Genet.*, **6**, 217–219.
BURKS RT, KESSIS TD, CHO KR AND HEDRICK L. (1994). Microsatellite instability in endometrial carcinoma. *Oncogene*, **9**, 1163–1166.
FIELD JK. (1992). Oncogenes and tumour-suppressor genes in squamous cell carcinoma of the head and neck. *Eur. J. Cancer Oral Oncol.*, **28B**, 67–76.
FIELD JK, SPANDIDOS DA, STELL PM, VAUGHAN ED, EVAN GI AND MOORE JP. (1989). Elevated expression of the c-myc oncoprotein correlates with poor prognosis in head and neck squamous cell carcinoma. *Oncogene*, **4**, 1463–1468.
FIELD JK, SPANDIDOS DA, MALLIRI A, YIAGNISIS M, GOSNEY JR AND STELL PM. (1991). Elevated p53 expression correlates with a history of heavy smoking in squamous cell carcinoma of the head and neck. *Br. J. Cancer*, **64**, 573–577.
FIELD JK, SPANDIDOS DA AND STELL PM. (1992). Overexpression of the p53 gene in head and neck cancer, linked with heavy smoking and drinking. *Lancet*, **339**, 502–503.
FIELD JK, TSIRIYOTIS C, ZOUMPOURLIS V, HOWARD P, SPANDIDOS DA AND JONES AS. (1994). Allele loss on chromsome 3 in squamous cell carcinoma of the head and neck correlates with poor clinical prognostic indicators. *Int. J. Oncol.*, **4**, 543–549.
IONOV Y, PEINADO MA, MALKHOSYAN S, SHIBATA D AND PERUCHO M. (1993). Ubiquitous somatic mutations in simple repeated sequences reveal a new mechanism for colonic carcinogenesis. *Nature*, **363**, 558–561.
KAPLAN EL AND MEIER P. (1958). Nonparametric estimation from complete observation. *J. Am. Stat. Assoc.*, **53**, 457–481.
KIARIS H, JONES AS, SPANDIDOS DA, VAUGHAN ED AND FIELD JK. (1994). Loss of heterozygosity on chromosome 8 in squamous cell carcinoma of the head and neck. *Int. J. Oncol.*, **5**, 1243–1248.
LINDBLOM A, TANNERGARD P, WERELIUS B AND NORDENSKJOLD M. (1993). Genetic mapping of a second locus predisposing to hereditary non-polyposis colon cancer. *Nature Genet.*, **5**, 279–282.
MERLO A, MABRY M, GABRIELSON E, VOLLMER R, BAYLIN SB AND SIDRANSKY D. (1994). Frequent microsatellite instability in primary small cell lung cancer. *Cancer Res.*, **54**, 2098–2101.
NICOLAIDES NC, PAPADOPOULOS N, LIU B, WEI Y, CARTER KC, RUBEN SM, ROSEN CA, HASELTINE WA, FLEISCHMANN RD, FRASER CM, ADAMS MD, VENTER JC, DUNLOP MG, HAMILTON SR, PETERSON GM, DE LA CHAPELLE A, VOGELSTEIN B AND KINZLER KW. (1994). Mutations of two PMS homologues in hereditary nonpolyposis colon cancer. *Nature*, **371**, 75–80.
PAPADOPOULOS N, NICOLAIDES NC, WEI YF, RUBEN SM, CARTER KC, ROSEN CA, HASELTINE WA, FLEISCHMANN RD, FRASER CM, ADAMS MD, VENTER JC, HAMILTON SR, PETERSEN GM, WATSON P, LYNCH HT, PELTOMAKI P, MECKLIN JP, DE LA CHAPELLE A, KINZLER KW AND VOGELSTEIN B. (1994). Mutation of a mutL homolog in hereditary colon cancer. *Science*, **263**, 1625–1629.
PELTOMÄKI P, AALTONEN LA, SISTONEN P, PYLKKÄNEN L, MECKLIN J-P, JÄRVINEN H, GREEN JS, JASS JR, WEBER JL, LEACH FS, PETERSEN GM, HAMILTON SR, DE LA CHAPELLE A AND VOGELSTEIN B. (1993). Genetic mapping of a locus predisposing to human colorectal cancer. *Science*, **260**, 810–812.
PETO R, PIKE MC, ARMITAGE PE, BRESLOW NE, COX DR, HOWARD SV, MANTEL N, MCPHERSON K, PETO J AND SMITH PG. (1976). Design and analysis of randomised clinical trials requiring prolonged observation of each patient. *Br. J. Cancer*, **34**, 585–612.
RICHARDS RI AND SUTHERLAND GR. (1994). Simple repeat DNA is not replicated simply. *Nature Genet.*, **6**, 114–116.
RHYU MG, PARK WS AND MELTZER SJ. (1994). Microsatellite instability occurs frequently in human gastric carcinoma. *Oncogene*, **9**, 29–32.
RISINGER JI, BERCHUCK A, KOHLER MF, WATSON P, LYNCH HT AND BOYD J. (1993). Genetic instability of microsatellites in endometrial carcinoma. *Cancer Res.*, **53**, 5100–5103.
SHIBATA D, PEINADO MA, IONOV Y, MALKHOSYAN S AND PERUCHO M. (1994). Genomic instability in repeated sequences is an early somatic event in colorectal tumorigenesis that persists after transformation. *Nature Genet.*, **6**, 273–281.
SHRIDHAR V, SIEGFRIED J, HUNT J, ALONSO MM AND SMITH DI. (1994). Genetic instability of microsatellite sequences in many non-small cell lung carcinomas. *Cancer Res.*, **54**, 2084–2087.
THIBODEAU SN, BREN G AND SCHAID D. (1993). Microsatellite instability in cancer of the proximal colon. *Science*, **260**, 816–819.
WOOSTER R, CLETON-JANSEN A-M, COLLINS N, MANJION J, CORNELIS RS, COOPER CS, GUSTERSON BA, PONDER BAJ, VON DEIMLING A, WIESTLER OD, CORNELISSE CJ, DEVILEE P AND STRATTON MR. (1994). Instability of short tandem repeats (microsatellites) in human cancers. *Nature Genet.*, **6**, 152–156.
YEE CJ, ROODI N, VERRIER CS AND PARL FF. (1994). Microsatellite instability and loss of heterozygosity in breast cancer. *Cancer Res.*, **54**, 1641–1644.